# What Influences Linkage to Care After Home-Based HIV Counseling and Testing?

**DOI:** 10.1007/s10461-017-1830-6

**Published:** 2017-06-22

**Authors:** Reshma Naik, Wanga Zembe, Fatima Adigun, Elizabeth Jackson, Hanani Tabana, Debra Jackson, Frank Feeley, Tanya Doherty

**Affiliations:** 1 Health Systems Research Unit, South African Medical Research Council, Tygerberg, South Africa; 2 Department of Global Health, School of Public Health, Boston University, Boston, MA USA; 3 Population Reference Bureau, 1875 Connecticut Avenue, NW, Suite 520, Washington, DC USA; 4 Heilbrunn Department of Population and Family Health, Mailman School of Public Health, Columbia University, New York City, NY USA; 5 Department of Public Health Sciences, Karolinska Institute, Stockholm, Sweden; 6 School of Public Health, University of the Western Cape, Bellville, South Africa

**Keywords:** Home-based, HIV counseling & testing, Linkage to care, HIV/AIDS, Test and treat, HIV care cascade, South Africa

## Abstract

To maximize the benefits of test and treat strategies that utilize community-based HIV testing, clients who test positive must link to care in a timely manner. However, linkage rates across the HIV treatment cascade are typically low and little is known about what might facilitate or hinder care-seeking behavior. This qualitative study was conducted within a home-based HIV counseling and testing (HBHCT) intervention in South Africa. In-depth interviews were conducted with 30 HBHCT clients who tested HIV positive to explore what influenced their care-seeking behavior. A set of field notes for 196 additional HBHCT clients who tested HIV positive at home were also reviewed and analyzed. Content analysis showed that linkage to care is influenced by a myriad of factors at the individual, relationship, community, and health system levels. These factors subtly interact and at times reinforce each other. While some factors such as belief in test results, coping ability, social support, and prior experiences with the health system affect clients’ desire and motivation to seek care, others such as limited time and resources affect their agency to do so. To ensure that the benefits of community-based testing models are realized through timely linkage to care, programs and interventions must take into account and address clients’ emotions, motivation levels, living situations, relationship dynamics, responsibilities, and personal resources.

## Introduction

Bold efforts to reduce the burden of the AIDS epidemic are increasingly a priority at both global and national levels. UNAIDS has set ambitious treatment targets known as “90–90–90”. By the year 2020, 90% of all people living with HIV will know their status, 90% of all people diagnosed will receive antiretroviral treatment (ART), and 90% of all people receiving ART will be virally suppressed [1]. Reaching these targets will require countries to invest in a variety of strategies. One such approach is “test and treat” whereby countries implement large-scale testing programs to identify HIV positive individuals, and then follow up with immediate treatment regardless of CD4 count [2]. If successfully implemented, it would yield reductions in rates of HIV transmission and death [3, 4].

To increase the number of individuals that know their HIV status, many sub-Saharan African countries are implementing a range of community-based testing models including home-based HIV counseling and testing (HBHCT). Current evidence suggests that HBHCT is a low cost, feasible, and highly acceptable intervention that effectively identifies HIV-positive individuals [2, 3, 5–24].

A systematic review of uptake in sub-Saharan African HBHCT programs shows that 58–99% of people accept HBHCT, and up to 79% of those testing positive are newly diagnosed [17]. This model identifies the highest percentage of HIV-positive individuals compared to standard or facility-based testing models [12]. Even though community testing models such as HBHCT often include post-test counseling and referrals to nearby HIV clinics, rates of linkage are typically not optimal. Studies define linkage differently and use different cutoff points, but linkage rates can range from as low as 14% up to 75% [2, 11, 13, 25–30]. There is a continual decrease in linkage at each stage of care from obtaining a CD4 count, determining eligibility for ART, and initiating ART [29, 31]. A 2017 Lancet paper reporting data from a longitudinal study in Kwazulu-Natal, suggests that the transition from testing to care is the weakest link in the HIV care cascade. Eight years following HIV testing, only 45% of patients had linked to care and the median time to linkage was 52 months [32]. Although the ‘test and treat’ approach will eliminate some of the delays in pre-ART care, clients can still be lost in the transition from the place where they test to an HIV treatment site and between an initial visit to an HIV treatment site and ART initiation. The reasons for sub-optimal linkage are not well understood and require further attention if global and national targets are to be achieved.

Nearly all studies that address the reasons for poor linkage are quantitative in nature. At the individual level, young clients and those with low socioeconomic status are less likely to seek care. Health systems factors such as long wait times or distance to clinics have also been found to be barriers [14, 25, 33, 34]. But, these factors do not appear to tell the full story. Two studies in South Africa show that even in a context where free HIV care and treatment services were provided close to HBHCT clients, linkage was not optimal. In one study, only 67% of participants began ART within 6 months [27]. In another, where HIV services were nearby and ART was offered regardless of CD4 count, fewer than 40% of HIV positive individuals linked to care within 3 months of HBHCT [35]. This indicates that there may be other more personal factors influencing linkage.

Some efforts to improve linkage both in facility-based and community-based settings include requiring less frequent clinic visits and medication pick-ups, and programs that provide community support [36]. In addition, removing CD4 count threshold criteria for ART could give clients assurance that linkage would result in treatment, as well as cut down the burdensome series of clinic visits that result in drop off. South Africa and other countries are, or soon will be, offering HIV treatment to all people living with HIV regardless of CD4 count [37]. While these approaches will go a long way to address important barriers, evidence from the broader HIV literature indicates that psychosocial and socio-cultural barriers likely also exist and similarly require solutions.

Systematic reviews on interventions to improve linkage and ART initiation show that evidence of what works is limited [38, 39]. There is an urgent need for strategies that increase rates of linkage to care, particularly within the context of community-based HIV testing. To inform the design of policy and program interventions to optimize ‘test and treat’, this qualitative study sought to uncover both facilitators and barriers to linkage among HIV-positive clients tested through a HBHCT program in rural South Africa. Using a socio-ecological framework, this paper presents and describes individual, relationship, community, and health system related factors, as well as how they interact and contribute to rapid, delayed, or failed linkage to care.

## Methods

### Study Design

This qualitative descriptive study was nested within a cluster randomized control trial (RCT) of HBHCT uptake conducted in Umzimkhulu (a poor, rural municipality of KwaZulu-Natal, South Africa) from September 2009 to January 2011. A detailed description of the RCT can be found elsewhere [6]. As part of the trial, community members in the intervention clusters were offered free rapid HIV testing in their homes by trained lay counselors if they were 18 years or older. Those aged 14–17 years could participate with parental or guardian consent. Approximately 9.7% (n = 492) of clients tested HIV positive. All HIV-positive clients were provided with post-test counseling and a referral letter to take to the clinic of their choice.

As part of a separate, but related quantitative sub-study also nested within the RCT, all consenting HIV-positive clients were followed up periodically by their counselors to monitor linkage to care, defined as providing a blood sample at a health facility for a CD4 count. Counselors used paper-based tracking forms to record clients’ progress with linkage to care. For those who linked, they recorded the name of the clinic, location, and date of visit. A nurse from the study team then visited clinics and checked official records to verify linkage. Due to protocol restrictions, this was only done at facilities within the study’s catchment area. For those who reported not linking to care, counselors continued to contact them until the end of the study. Each time, they recorded the date of that self-report. The full set of procedures for tracking and documenting linkage are described elsewhere [14].

Time from the date of testing to the date of the CD4 was calculated for all HIV-positive clients who linked to care. Consistent with recommended cutoffs for this stage of pre-ART care, prompt linkage was defined as linkage within 3 months of testing [40]. For those who did not link, we calculated the time from testing to the last known point of contact when non-linkage was reported.

Approximately 62% of the 492 HIV-positive HBHCT clients linked to care within 3 months of testing. The remaining clients linked to care after 3 months, had not linked at the last point of contact, or were lost to follow up [14].

### Qualitative Data Collection

To understand clients’ reasons for timely, delayed, or non-linkage to care, we conducted in-depth interviews (IDI) with a purposive sample of 30 of the 492 HIV positive HBHCT clients selected based on their documented care-seeking behavior (prompt linkage, delayed linkage, or no linkage). The study investigator reviewed documentation of time to linkage and drew up lists of potential clients who could be interviewed in each of the three categories. Those with verified records were prioritized. The interviewer then contacted clients to ask if they would be willing to participate in an in-depth interview. Five clients refused because they were not comfortable with a follow-up visit; many other clients on the list were difficult to reach, even after multiple tries. Several clients were contacted before we had enough IDI participants who were representative of each of the three linkage categories.

Interview participants were drawn from 12 of the 19 intervention clusters to reflect the potential differences in the distance to and characteristics of the closest clinic in the catchment area. Of the 30 clients interviewed, a total of 21 linked to care at some point during the study. This included 14 clients with verified clinic records who sought care within 3 months of testing and 7 who delayed and linked within 107–520 days of testing. The other 9 clients interviewed had not linked to care by the last point of contact, which ranged from 123 to 542 days following HBHCT.

All IDIs were conducted using a semi-structured interview guide by a local, female interviewer in a mixed local dialect of Zulu and Xhosa. The guide included open-ended questions to explore experiences with HBHCT and follow-up behavior. The interviewer either approached participants in their homes or called participants to determine a time they could be met in their homes or elsewhere. Each participant who provided informed consent was interviewed in a private room or area of the home. Each interview lasted about 35 minutes and was recorded using a digital audio recorder.

To add further context and richness, we also reviewed and analyzed 196 field notes that were taken as part of the above mentioned quantitative study on linkage that was also nested within the main RCT [14]. The field notes were taken prior to administration of a survey among HIV-positive HBHCT clients. The interviewer had been instructed to record any information the client shared about their reasons for either linking or not linking to care. Typically, these notes were a few sentences to a paragraph long. Although we did not initially plan to include the field notes as data for this study, upon reviewing them, the investigator felt strongly that the content was informative and complemented the in-depth interviews.

### Data Preparation

The audio files were transcribed verbatim in their original languages by two transcriptionists separate from the study team. They were then translated into English by the same individuals. To monitor transcription and translation quality, a senior member of the research team fluent in Zulu and Xhosa, as well as in English, cross-checked a sample of interviews throughout the process and provided feedback to the transcriptionists/translators. No major discrepancies were identified. The handwritten field notes were typed verbatim in English.

### Data Analysis

The authors chose a socio-ecological model (SEM) to guide data analysis. This model was chosen as it is a theory-based framework for understanding the multifaceted and interactive effects of personal and environmental factors that determine behaviors, and for identifying behavioral and organizational (health system) leverage points and intermediaries for improving health behavior. There are four nested, hierarchical levels of the SEM used in this analysis: individual, interpersonal/relationships, community, and health system [41].

1. The investigator read through all 30 in-depth interview transcripts and 196 field notes, reflected on key themes, and developed an outline of the primary themes emerging from the data according to the four SEM levels. A second reader separate from the study team, read through a 20% sample of in-depth interview transcripts (n = 6) and a 10% sample of field notes (n = 20), and independently developed an outline of recurrent themes. The investigator and second reader then discussed the thematic outlines, reflected on key concepts, clarified emerging ideas, and identified new patterns. Based on this, the investigator developed a new outline and created a codebook broadly outlining key concepts. The codebook was discussed and further modified with the second reader. Qualitative software, Atlas.ti version 7.0.71, was then used to code, sort, and categorize the data. A very important part of the analysis was an exploration of the patterns that emerged when the qualitative findings and time-to-CD4 data were paired. We identified similarities in characteristics, attitudes, or experiences that were common among clients who linked to care quickly, slowly, or not at all. Key themes were written up and refined after further discussions with the second reader as well as the broader research team. The findings that emerged from this process are presented and described as results.

## Results

Among the 30 clients who participated in an in-depth interview, 24 (80%) were female and 6 (20%) were male, reflecting the underlying population. Characteristics of these participants were similar to all HIV-positive clients identified by HBHCT (Table 1). Note the high proportion of females is expected for this setting and is further described elsewhere [42].

**Table 1:** Characteristics of clients included in the qualitative analysis

Characteristic	All HIV-positive HBHCT clients (N = 492) n (%)	Clients who participated in the in-depth interviews (N = 30) n (%)
Household size		
<3 adult members	305 (62.0)	20 (66.7)
≥3 adult members	187 (38.0)	10 (33.3)
Gender		
Male	101 (20.5)	6 (20.0)
Female	391 (79.5)	24 (80.0)
Age group		
16–24	110 (22.4)	6 (20.0)
25–49	303 (61.6)	18 (60.0)
50+	79 (16.1)	6 (20.0)
Marital status		
Single	220 (44.7)	12 (40.0)
Married/co-habitating	202 (41.1)	13 (43.3)
Divorced/separated/widowed	70 (14.2)	5 (16.7)
Ever had an HIV test before HBHCT		
Yes	265 (53.9)	14 (46.7)
No	227 (46.1)	16 (53.3)

**Fig. 1:**
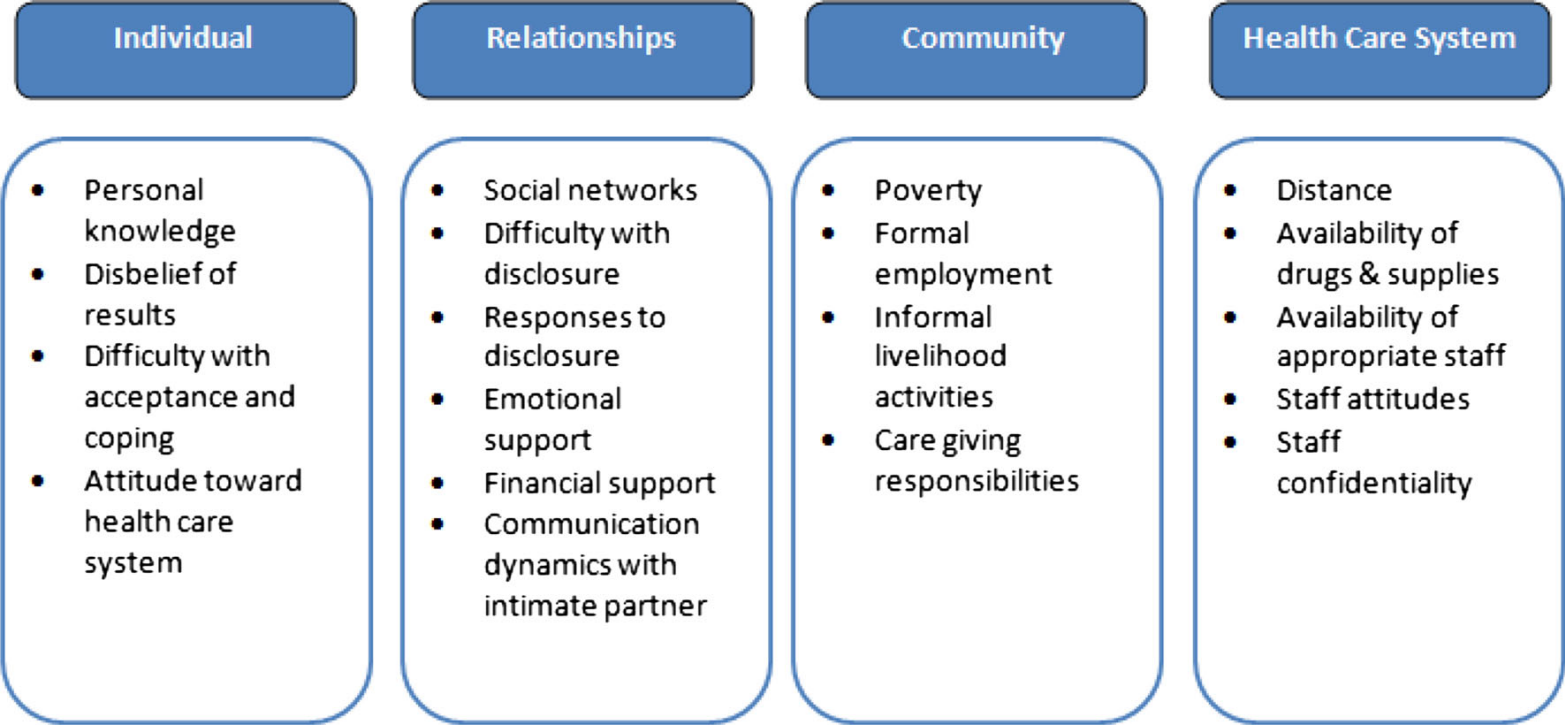
Socio-ecological factors influencing linkage to care

The analysis showed that linkage to care is affected by a variety of factors within the four levels of the SEM, and that these all operate in concert. Clients each have a unique set of circumstances, which can include both potential barriers and facilitating factors. However, these factors do not necessarily hinder or encourage linkage, respectively. How the factors combine and interact is what ultimately drives behavior. Despite this complexity, the findings have been organized using the four levels of the SEM: individual, relationships, community, and health care system. Figure 1 shows the full list of themes that emerged. The most prominent ones are summarized and further explained below, supported by illustrative quotes from the in-depth interviews.

### Individual

At the individual level, findings highlight that clients’ responses to the diagnosis, coping abilities, and personal beliefs about the health system affected linkage. When pairing the time-to-CD4 data with the qualitative findings, clear differences emerged between those who delayed care seeking and those who sought care quickly. Those who delayed, typically struggled to believe and accept their diagnoses, worried excessively, or had an attitude reflecting nonchalance, apathy, or futility. These quotes illustrate some of the commonly expressed responses, emotions, and attitudes experienced by those who significantly delayed linkage.

One client said she found it very difficult to accept her diagnosis and had turned to drinking to cope:

I drink the whole week and I even see that people can see that I’m now crazy because from early in the morning around 8 or 9AM, I have started drinking. I drink and drink so that I may just forget a bit.—Female, 38 years, had not linked in 226 days

Another struggled to believe the results:

No sister, I don’t believe [the test result] and I still don’t believe it even today… where did I get it because I’m a widow and I don’t go around taking other women’s men but then where did I get it?—Female, 47 years, had not linked to care at the last point of contact (231 days)

This client described her fears to the interviewer:

Interviewer: …you mentioned among other things that one of the reasons you didn’t take the letter to the clinic is that you have fear about going there. Can you explain to me the cause of this fear?

Respondent: …it is just scary because a person who is infected can just die, those are the things that scare me.—Female, 36 years, had not linked at last point of contact (123 days)

When asked why he did not seek care, one client responded:

…it was just laziness….it’s just that they are going to keep on telling me about this issue you see. They are going to tell me that I must go to the clinic and come back all the time. So now I just feel lazy to go there.—Male, 32 years, had not linked to care at last point of contact (244 days)

On the other hand, those who linked promptly typically expressed acceptance of their situation, and a keen sense of motivation and agency. Many such clients had also expressed having a strong interest in testing as they had suspected something was wrong and were eager to get help. These quotes illustrate how acceptance can influence care seeking:

Because we managed to accept our situation, our status, I think that is what helped us to go [to the clinic] quickly. So that we can know how far are we with our health status as a whole.—Male, 27 years, linked to care in 5 days

I was encouraged by the condition that I had learnt that I’m in so that they can see how much my CD4 count was so that they can see what they can do…I must not sit and accept this thing…I quickly went [to the clinic] because I had learnt that I have the virus.—Female, 41 years, linked to care in 5 days

### Relationship

Factors at the relationship level also affected clients’ responses to the diagnosis and their care-seeking behavior. In particular, those who lacked close confidants and support, especially within their own households, typically delayed care seeking. It was commonly mentioned in the field notes that clients struggled with disclosure. One participant who had still not sought care several months after testing talked about the possibility of telling her grandmother as she felt she would be the most understanding, but since her grandmother lived far away, this was difficult. Other clients felt they had to wait for the right timing or await the return of their partners who were away for extended periods before they could disclose. Some who did disclose received negative, discouraging responses, while others feared such responses and being blamed for their illness. One of the field notes describes a situation in which a client who delayed seeking care more than 6 months was berated by her partner after disclosing her test results.

The following quotes relating to clients who delayed linkage illustrate challenges with disclosure. Difficulty disclosing to those within the household is a particular challenge as it could make it especially hard to find opportunities to seek care without raising suspicions.

…I would have told my friend but I’m not going to tell anyone here in the household. I don’t want to lie I will never. People from this household are talkative. This will make me to be always embarrassed because I know how the situation is here in the household.—Female, 24 years, linked to care in 167 days

I will indeed go [to the clinic] but I’m still waiting for my husband and we will go together as well as talk about it. I won’t say anything on the phone. Things like this you can’t just say it anyhow…. I’m not saying that I won’t do it but my stand is, we need to go together.—Female, 32 years, had not linked to care at last point of contact (542 days)

In contrast, many who linked to care quickly talked about the emotional and financial support they received from family, friends, intimate partners, and the counselor. In the field notes, some clients mentioned that their partners were very caring, providing money and encouragement to seek care. One particular client who linked to care within a month mentioned she tested together with her husband and he was very understanding and is the only one who knows her status; they both felt comforted by the counselor who gave them a sense of hope and optimism. These quotes from clients who promptly linked to care show the importance of social support:

My sister is giving much support, she is like a parent to me, she is just like my mother.—Female, 39 years, linked to care in 2 days

Something that made me to go [to the clinic] is that I kept on meeting [the counselor] on the way and each time we meet on the way she will ask me…asking if I have been to the clinic and I will say no I haven’t been and she will say she’s getting worried. You see each time she says that I will just say let me just hand myself over and just go…I just went then…it’s that lady, I kept on meeting her and she will encourage me such that she will go to my place and ask me to go outside and discuss this…she just kept on encouraging me.—Female, 29 years, linked to care in 15 days

[My girlfriend] didn’t take it badly, she never had a problem.—Male, 32 years, linked to care in 8 days

### Community

Community level factors related to the poor and rural setting clearly affected care-seeking behavior. Most clients faced extreme financial hardships and were burdened with various household and care-giving responsibilities. Clients also spoke about having to spend extensive amounts of time and energy searching for work or engaging in livelihood activities. The key theme that emerged is that even when clients strongly desired to seek care, they often delayed because caregiving responsibilities, job seeking, or other circumstances were a higher priority and limited their time and opportunities to attend a health care facility. The following quotes illustrate this concept:

…it took me some time [to go to the clinic] because… I have been busy, not having time…I have been busy my sister, I have been walking up and down looking for a job as you can see that I am unemployed.—Male, 56 years, linked to care in 213 days

…I’ve been staying with my grandmother the whole day….my mother gets off only on Saturdays, she works from Monday to Saturday. On Saturday, she knocks off at 3 pm and clinics don’t operate on Sundays. That’s the thing that has been making me not be able to go to the clinic.—Female, 20 years, had not linked to care at last point of contact (146 days)

My sister I have been poor. I have been having problems sister. Whatever I have been getting I’ve been getting through hard work. I had to do some work before getting money. I was not going to take that money and go to [name of health facility] when my children don’t have food you see that.**—**Female, 47 years, had not linked to care at last point of contact (231 days)

### Health System

Several clients described challenging experiences with the health care system including long wait times, being turned away, lack of appropriate supplies, and poor staff attitudes or breaches of confidentiality. A combination of these previous experiences and knowing the extensive time and financial burdens involved in seeking care discouraged even those clients who desired to seek care and did not face other barriers. Some of the field notes described situations where clients did attempt to seek care and were turned away because the clinic did not have needles or the right lab forms, or staff available to take a blood sample. Such clients were typically reluctant to try again.

The following quotations illustrate how even motivated clients can feel disillusioned and delay care seeking due to their expectations of cumbersome, challenging, and frustrating experiences with the health care system:

[The clinic] becomes too full, sometimes you will go there and nothing will be done to you because there are a lot of people. Sometimes by the time you get there the gate is closed and they will say they only want 50 people. You have to wake up while it’s still dark and go to the clinic. By the time they open at 8am you are already waiting at the gate.—Female, 48 years, linked to care in 75 days

You just lose hope because you come from far…you come dragging yourself because you also don’t like what you are there to do, it’s just that you have to. Then you get there and find that there is no one that encourages you. No, the person who checks results is not there, no, we don’t draw blood today, no, we are busy, you see that?—Female, 29 years, linked to care in 131 days

I haven’t done anything with [the referral letter]…because staff at the local clinic don’t have confidentiality…I’ve heard them talking about other people… I have to use money to go to the clinic that I like but then I don’t have money yet.—Female, 22 years, had not linked at last point of contact (192 days)

One client described her failed attempt to seek care:

[The nurse] told me to go and join the queue and it was full…I could see that time was against me and there was no transport and there was nothing that I was sitting there for. That is when I came back with that [referral] letter that later got lost and burnt with the goods.—Female, 26 years, had not linked at last point of contact (521 days)

## Discussion

This study is among few qualitative studies to explore linkage to care following community-based HIV testing. Although the general views and experiences of clients in this study are not new to the HIV/AIDS literature, this analysis does underscore their importance when it comes to facilitating or hindering linkage. Although the findings were categorized into four levels it was apparent that no one factor alone served as a primary barrier or facilitator of linkage. Rather, any given client’s care-seeking behavior was influenced by a combination of interacting factors and life circumstances. While some factors like disbelief of the test results affected the desire to seek care, others such as caregiving responsibilities affected clients’ sense of empowerment and agency to do so.

For each client, the various individual, relationship, community, and health systems factors subtly interacted, sometimes reinforcing each other, and ultimately driving behavior. In some cases, the combination of various difficult circumstances ultimately hindered linkage, while in others, “just enough” positive circumstances facilitated it. Motivation levels and positive coping do seem to play a particularly significant role in how resilient people are in facing various barriers. For example, for a person who is only mildly motivated, negative past experiences with the health care system combined with heavy family responsibilities might be enough to deter them from seeking care. In contrast, a highly-motivated individual who also has social support may be more willing and able to try to overcome financial, logistic, or other barriers to care. A combination of the “right” factors, different for each person, is needed to ultimately drive timely linkage.

The findings complement and give context and richer understanding to what we already know from the literature and programmatic experience about how individual, relationship, community, and health systems factors influence care seeking behavior. Hearing the “voices” of clients provides insight into their inner lives, and provides an opportunity to better understand their needs. Belief and acceptance of results, as well as positive coping have been identified as important factors [11, 14, 34] for care seeking; these findings elucidate some of the reasons underlying those challenges. Similarly, our findings address the important topic of disclosure, which is known to affect linkage [24]. Who is disclosed to, and how they respond, has a strong influence on care-seeking behavior. The ability of clients to disclose to and receive support from those with a more proximate or strong influence on their daily lives, is more likely to facilitate care. Meanwhile those who cannot bring themselves to disclose, who wish to wait, or who receive negative responses may feel disempowered to further address their diagnoses through care seeking.

Logistic barriers to care seeking such as long distances, and lack of time and finances are commonly discussed in the health care and HIV/AIDS literature. These qualitative findings suggest that such factors may not necessarily be firm constraints, but rather contextual elements that subtly push care seeking further down on the list of personal priorities. It may not necessarily be an issue of lack of money or hours in the day, but about hardships that make it difficult to spend such limited resources on care seeking. These challenging circumstances may also further discourage those who are already lacking motivation or support to address an HIV diagnosis.

Various studies on health services utilization in sub-Saharan Africa have cited factors such as frequent staff shortages, poor health worker attitudes and practices, poor quality of care, and drug and supply stock outs, to be important barriers to health services utilization in general [43, 44]. The literature on barriers to HIV testing, ART adherence, and HIV care seeking in sub-Saharan Africa also notably cites negative experiences with clinic staff as important barriers [45–49]. What this qualitative study showed is that these experiences are strongly internalized and can dampen clients’ inclination to seek care even for other health concerns in the future. Addressing these barriers will be critical in the current era of universal test and treat and self-testing, the impact of which depends on timely linkage to care.

It is reasonable to extrapolate from our findings that stigma played an important role in influencing linkage to care at each level of the socio-ecological framework. Clients may have had difficulty with acceptance and coping due to fears of embarrassment or isolation. At the relationship level, these fears may have affected the willingness to disclose to others, and in turn stigma may have led to negative responses to disclosure rather than encouragement and support. Finally, the discomfort clients feel when seeking care at clinics and concerns about lack of confidentiality may also stem from stigma.

### Strengths and Limitations

This study has a number of important strengths. To our knowledge, it is one of the first qualitative studies to date that explores linkage to care following HBHCT. Its placement within a randomized controlled trial enabled a real-time investigation, which may have allowed clients to better recall and more accurately describe the most relevant and current circumstances in their lives related to testing and follow up. Further, this study took place in a setting with a relatively poor rural population, broad geographic area and care provided by government clinics with no additional support from the study. Thus, the factors identified can be considered to represent experiences typical of clients in a poor rural South African context. Third and quite importantly, this study addresses psychosocial factors impacting late presentation for HIV/AIDS services, which are less well understood and difficult to assess using only quantitative research methods. Fourth, the purposive sampling and pairing of actual time-to-CD4 data with qualitative information, provided a very powerful illustration of the salient characteristics and factors that are likely to have influenced linkage to care. In particular, this method enabled us to identify patterns surrounding attitudes and personal relationships that clients themselves were not necessarily able to articulate. Finally, the findings from this study are likely to be broadly applicable to settings outside of home-based testing, and to various points of linkage along the continuum of engagement in HIV care.

Alongside its strengths, the study also has some important limitations. First, it should be acknowledged that the topic of the interviews was potentially sensitive for clients and may have been difficult to process and discuss. Although care was taken to undertake interviews in a private venue with a well-trained interviewer, the findings may not reflect some barriers which may be too difficult to verbalize or acknowledge. Second, since this study took place in one rural South African community with a primarily female resident population, the findings cannot be broadly generalized. However, the findings and principles are transferable to similar populations and settings in South Africa as well as other countries in the region. Further, given that many unique challenges to linkage may result from testing women in the absence of their male partners, this study offers valuable insights.

## Conclusion

Our study identifies facilitators and barriers to linkage to care following HBHCT at each level of the socio-ecological model. Since these factors can interact and reinforce each other, approaches to address them must be holistic, addressing the full spectrum of client’s lives, including emotions, motivation levels, living situations, relationship dynamics, responsibilities, and personal resources. Approaches that may help clients adjust to and cope with their diagnosis, disclose, and move forward with care and treatment include tailored counseling, short-term post-testing support or wellness groups, or brief disclosure interventions. Encouraging couples testing and assessing and addressing clients’ prospects for social support at the point of testing could also help facilitate linkage.

Another way to support clients would be to integrate monitoring of linkage to care into the role of community health workers or lay counselors. Counselors could act as a paraprofessional patient navigator and provide intensive outreach to clients who are facing exceptional challenges and who have not promptly engaged in care. Innovative service delivery models, such as home-based ART initiation, that bring health care to people’s homes could also help overcome some of the logistic barriers. This study also highlighted the need for broader systems level changes such as addressing negative attitudes of health workers towards clients and providing more client friendly services. Since stigma likely underlies many of the challenges that clients face when it comes to linkage, anti-stigma interventions could be implemented in concert with community-based HIV testing initiatives.

Overall, these findings can inform programmatic strategies in South Africa and other countries that are implementing a ‘test and treat’ approach. Although this study defined linkage as getting a CD4 count—a step in the cascade that will be removed within ‘test and treat’—the same challenges are still likely to apply for those who are tested within home- or other community-based testing models and who need to be promptly linked to a facility for treatment counseling and initiation. Many improvements to community-oriented HIV testing interventions could help address these challenges and optimize outcomes. Among these are broad systems level changes, and focused efforts to promote linkage to care that consider the full context of clients’ lives.
